# Plasma gelsolin as a potential biomarker for intrauterine inflammation in pregnant women with preterm premature rupture of membranes: A pilot study

**DOI:** 10.1371/journal.pone.0346499

**Published:** 2026-04-07

**Authors:** Yumiko Miyazaki, Yuko Fujita, Masayuki Fujita, Toshimichi Onuma, Hideaki Tsuyoshi, Makoto Orisaka, Yoshio Yoshida

**Affiliations:** Department of Obstetrics and Gynecology, Faculty of Medical Sciences, University of Fukui, Fukui, Japan; Korea University - Seoul Campus: Korea University, KOREA, REPUBLIC OF

## Abstract

**Background:**

Gelsolin is an actin-binding protein, the blood levels of which decrease in response to inflammation and tissue injury. However, the dynamics of gelsolin during pregnancy and its relationship with intrauterine inflammation remain unclear. In cases of preterm premature rupture of membranes, conventional indicators such as white blood cell (WBC) count and C-reactive protein (CRP) have known limitations, and reliable biomarkers for predicting intrauterine inflammation are limited. In this study, we investigated changes in plasma gelsolin concentrations during pregnancy and evaluated their association with intrauterine inflammation in patients with preterm premature rupture of membranes.

**Methods:**

In this pilot study, plasma gelsolin concentrations were measured by enzyme-linked immunosorbent assay in healthy pregnant women and in patients with preterm premature rupture of membranes. In healthy pregnancies, samples from the first, second, and third trimesters were analyzed. In cases with preterm premature rupture of membranes, serial measurements were performed from the time of membrane rupture to delivery. Correlation analyses with inflammatory markers (C-reactive protein and white blood cell) were conducted. Additionally, immunohistochemical staining for gelsolin was performed on placental tissues from chorioamnionitis cases and controls to compare expression levels and distribution patterns.

**Results:**

In healthy pregnant women, plasma gelsolin levels significantly decreased as pregnancy progressed. In cases with preterm premature rupture of membranes, plasma gelsolin levels showed a significant negative correlation with C-reactive protein and tended to decrease over time following membrane rupture. Immunohistochemical staining revealed a trend toward an increased number of gelsolin-positive cells in the chorionic membrane of chorioamnionitis cases.

**Conclusion:**

Plasma gelsolin decreases with the progression of pregnancy and intrauterine inflammation and may particularly reflect the progression of intrauterine inflammation in cases of preterm premature rupture of membrane. Gelsolin has the potential to serve as a novel biomarker for detecting inflammatory conditions that are not readily identified by conventional markers.

## Introduction

The intrauterine environment during pregnancy is a unique state that requires a balance between fetal development and maternal immune regulation. Dysregulation of the inflammatory response can lead to preterm birth and adverse fetal outcomes. Chorioamnionitis (CAM) is an inflammatory disorder caused by intrauterine infection or sterile inflammation and is recognized as a major cause of preterm premature rupture of membranes (pPROM), preterm birth and fetal inflammatory response syndrome (FIRS) [1]. Several markers of intrauterine infection—such as elevated concentrations of fibronectin in cervical or vaginal secretions, cervical shortening, and increased serum levels of granulocyte colony-stimulating factor and ferritin—have been associated with an increased risk of spontaneous preterm birth. However, the utility of these markers in reducing preterm birth risk or informing pregnancy-management strategies has not been demonstrated [2]. Furthermore, while CAM has traditionally been regarded as an infection-driven condition, recent attention has also focused on the concept of “CAM without overt infection,” in which subtle immune responses or maternal cytokine signaling may contribute to the pathophysiology [3]. These findings suggest that conventional infection markers alone may be insufficient for early detection or stratification of inflammatory conditions in pregnancy, including CAM.

Gelsolin (GSN) is a key regulator of cytoskeletal remodeling, functioning by severing and capping intracellular and extracellular actin filaments [4]. It plays a crucial role in various cellular processes, including cell motility, secretion, and apoptosis [5]. Plasma GSN (pGSN), the extracellular form circulating in the blood, clears extracellular actin released during tissue injury [4]. In addition, it modulates inflammatory cytokines and neutralizes proinflammatory molecules such as lipopolysaccharides [5]. Thus, pGSN is not merely a structural protein, but is considered a central component of the “actin scavenger” system, contributing to immune homeostasis and inflammation regulation. pGSN levels decrease during the acute phase of sepsis and traumatic injury, reflecting its consumption under such pathological conditions [5,6]. Regarding the regulation of pGSN during pregnancy, previous studies have shown in mouse models that pregnancy-related hormones, such as progesterone and human chorionic gonadotropin, promote the release of GSN into the plasma [7]. Additionally, a decrease in pGSN levels has been reported in inflammatory conditions such as hypertensive disorders of pregnancy, including preeclampsia [8,9]. However, there have been few reports focusing on longitudinal changes in pGSN or its placental expression in cases of pPROM and CAM. In this study, we preliminarily investigated changes in pGSN concentrations throughout pregnancy, the temporal dynamics of pGSN following pPROM, and the localized expression of cytosolic GSN (cGSN) in CAM. Based on these findings, we explored the potential utility of GSN as an adjunctive biomarker for predicting preterm birth and CAM following pPROM.

## Materials and methods

### Participants and sample collection

To evaluate changes in pGSN concentrations during pregnancy, maternal blood samples were collected from pregnant women who received perinatal care at our hospital between April 2023 and March 2025. A total of 66 women were enrolled in this study, including 8 non-pregnant volunteers (control group) and 58 pregnant participants. Pregnant participants were categorized into two groups: healthy pregnant women (n = 50), who provided blood samples in the first (11–13 weeks, n = 14), second (23–27 weeks, n = 18), and third (34–38 weeks, n = 18) trimesters, and women with preterm premature rupture of membranes (pPROM) (n = 8), who provided serial blood samples longitudinally from the time of membrane rupture (defined as day 0) until delivery. These samples were collected from −2–0 days relative to ROM and from −6–0 days relative to delivery, depending on clinical circumstances. The number of available samples varied among cases. All eight participants received prophylactic antibiotic therapy for 7 days after membrane rupture.　As this was a pilot study aimed at assessing longitudinal changes in pGSN levels during pregnancy, the sample size was determined based on the feasibility of participant recruitment and sample collection within the study period rather than by a formal power calculation. pGSN concentrations were measured by enzyme-linked immunosorbent assay. After delivery, placental and amniotic membrane tissues were collected for pathological examination and immunohistochemical analysis. Data access for research purposes occurred between 22 April 2025 and 25 June 2025. The authors had access to personally identifiable information, such as patient names and hospital IDs, during and after data collection. However, all data were handled in accordance with institutional ethical guidelines, and the study was approved by the Ethics Committee of the University of Fukui (approval number: 20250008). To ensure confidentiality, all data were anonymized prior to analysis. The requirement for written informed consent was waived, and an opt-out consent procedure was implemented. Information about the study was posted on the hospital website, and participants were given the opportunity to decline participation.

### Enzyme-linked immunosorbent assay (ELISA)

All blood samples were collected in ethylenediaminetetraacetic acid tubes, and plasma was isolated and stored at −20 °C until analysis. pGSN concentrations were assayed by ELISA (Aviscera Bioscience Inc., CA), according to manufacturer’s instructions. Plasma samples were diluted in a sample buffer (1/5000; Aviscera Bioscience, Inc. CA) and all analyses were duplicated. Optical densities were determined using a microtiter plate reader at 450 nm. Interleukin-6 (IL-6) concentrations were measured by ELISA (QK206, R&D Systems Inc., MN) using plasma diluted at 1:2, and tumor necrosis factor-alpha (TNF-α) levels were measured in undiluted plasma by ELISA (QK210, R&D Systems Inc., MN), according to the manufacturer’s instructions.

### Immunohistochemistry and placental pathology

We collected the placentas and umbilical cords after delivery. Sections were fixed in 10% neutral-buffered formalin, embedded in paraffin, and sectioned at a thickness of 3 µm. Sections were stained using an anti-GSN antibody (ab75832, Abcam, Cambridge, UK). Ten fields per section were examined under 200 × magnification to assess the localization and distribution patterns of cytosolic GSN-positive cells. Histological diagnoses of CAM and funisitis were determined by the degree of neutrophilic infiltration in stages I, II, and III based on the Blanc classification and the Nakayama classification, respectively [10,11].

### Biostatistical methods

Comparison of pGSN levels by gestational age was performed using the Kruskal–Wallis test. In 24 women who underwent spontaneous vaginal delivery between 35 weeks + 2 days and 37 weeks + 3 days of gestation, pGSN levels and the number of days until delivery were assessed. The predictive ability of pGSN for delivery within 28 days was evaluated using receiver operating characteristic (ROC) curve analysis, and the area under the curve (AUC) was calculated. To evaluate the relative decrease in pGSN, the rate of pGSN decline was defined as (pGSN at ROM − pGSN at delivery) / pGSN at ROM. Temporal changes in white blood cells (WBCs), C-reactive protein (CRP), and pGSN from rupture of membranes to delivery in pPROM cases were assessed using the Wilcoxon signed-rank test. Correlations between pGSN and other inflammatory markers (WBC and CRP) were analyzed using Spearman’s rank correlation coefficient. Intergroup comparisons of positive cell counts by immunohistochemistry were performed using the Mann–Whitney U test. All analyses were conducted using GraphPad Prism 10 (GraphPad Software Inc, San Diego, CA) and EZR software version 1.68, with a significance level set at *p* < 0.05.

## Results

### Changes in pGSN levels during pregnancy

**Fig 1:**
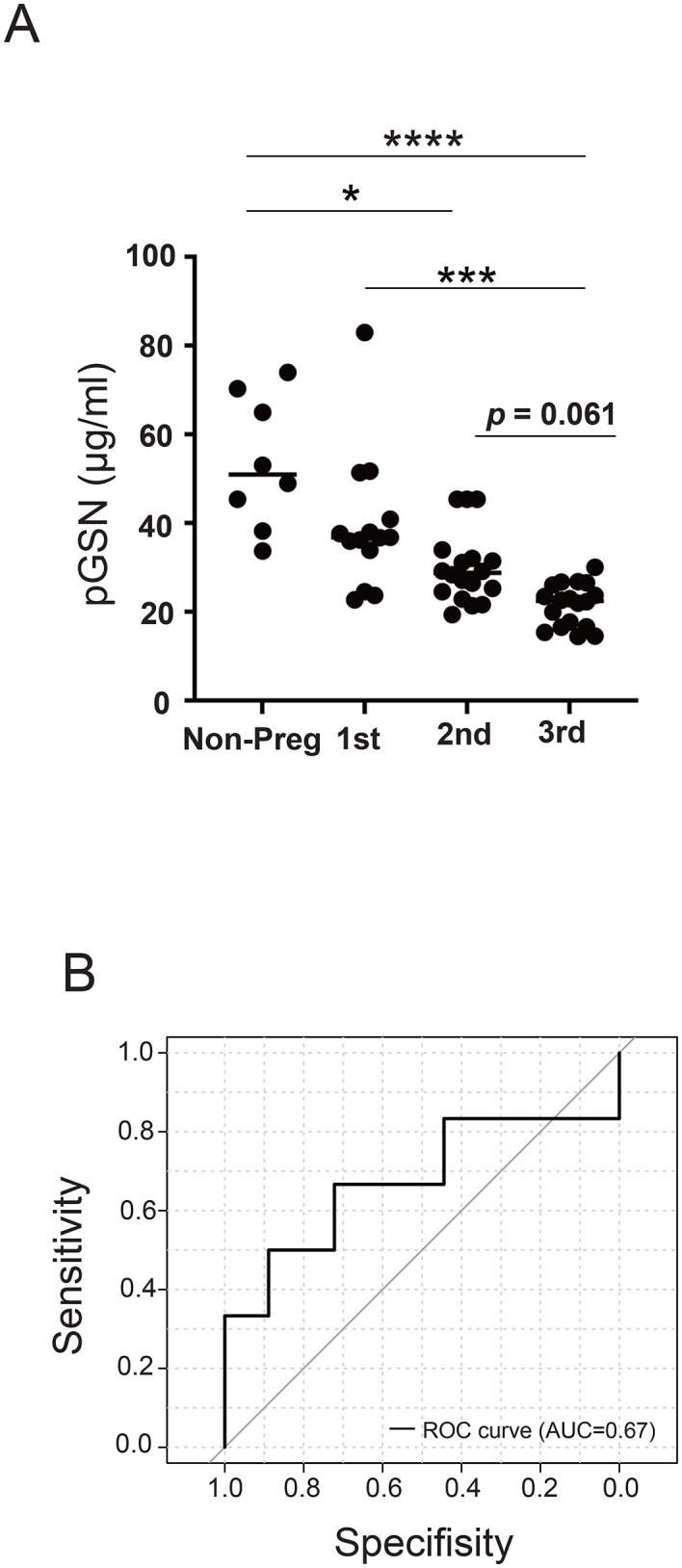
pGSN levels during pregnancy and prediction of delivery within 28 days. (A) Plasma gelsolin (pGSN) levels in samples from pregnant women during the first, second, and third trimesters, as well as non-pregnant (Non-Preg) women. The mean ± SD pGSN concentrations were 53.54 ± 14.84 μg/mL in non-pregnant controls (n = 8), 39.49 ± 15.22 μg/mL in normal pregnancies during the first trimester (n = 14), 30.00 ± 8.11 μg/mL in the second trimester (n = 18), and 21.57 ± 4.77 μg/mL in the third trimester (n = 18). The mean ± SD pGSN concentrations significantly decreased across gestational stages (one-way ANOVA, *p* < 0.0001). Post hoc comparisons revealed significant differences between non-pregnant controls and the second trimester (*p* = 0.013), non-pregnant controls and the third trimester (*p* < 0.0001), and between the first and third trimesters (*p* = 0.0002). Bars represent median values. * *p* < 0.05; *** *p* < 0.005; **** *p* < 0.0001. (B) Receiver operating characteristic (ROC) curve for the prediction of delivery within 28 days using pGSN levels. The area under the ROC curve (AUC) was 0.67.

In healthy pregnant women, pGSN concentrations were significantly lower than those in non-pregnant controls. Furthermore, pGSN levels in the third trimester were significantly decreased than that in the first trimester (Fig 1A). Although pGSN levels in the third trimester tended to be lower than those in the second trimester, the difference did not reach statistical significance (*p* = 0.053, Fig 1A). Among healthy pregnant women at 35–37 weeks of gestation, no statistically significant correlation was observed (S1 Fig), suggesting that pGSN alone may not predict the timing of spontaneous labor in late pregnancy. ROC curve analysis showed that the AUC for predicting delivery within 28 days using pGSN was 0.676, indicating only limited clinical utility in predicting delivery timing (Fig 1B).

### Temporal changes in GSN and its relationship with inflammatory markers in pPROM cases

**Fig 2:**
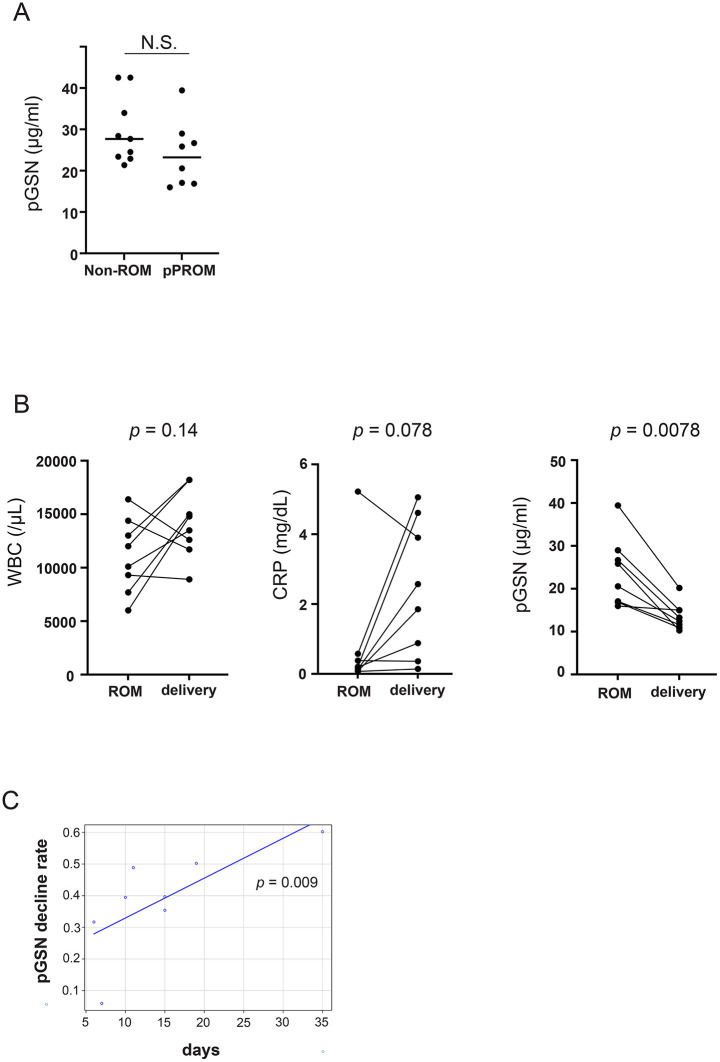
Temporal changes in WBC, CRP, and pGSN levels in a patients with pPROM. (A) Plasma gelsolin (pGSN) levels at the time of membrane rupture (day −2 to day 0) in cases of preterm premature rupture of membranes (pPROM), compared with levels in pregnant women without membrane rupture (Non-ROM) during the second trimester (25–33 weeks of gestation). No statistically significant difference in pGSN levels was observed between the two groups (*p* = 0.16). (B) Comparison of WBC, CRP, and pGSN levels at the time of membrane rupture (ROM; day −2 to day 0) and at delivery (0–7 days before delivery) in patients with pPROM. pGSN levels were significantly lower at delivery compared to the time of membrane rupture. (C) Correlation between the rate of pGSN decline and the interval from ROM to delivery (Spearman’s ρ = 0.84, *p* = 0.009). WBC, white blood cell; CRP, C-reactive protein; ROM, rupture of membrane.

**Fig 3:**
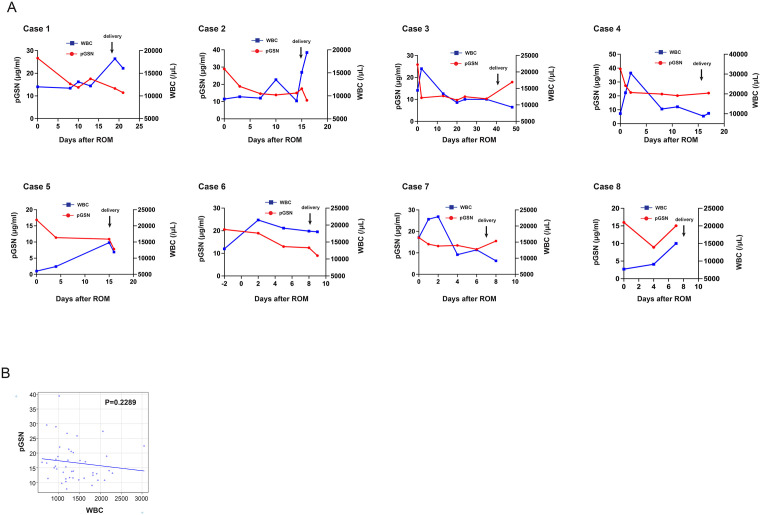
Temporal changes and correlation between pGSN and WBC in pPROM cases. (A) Longitudinal changes in pGSN levels and white blood cell (WBC) counts in individual pPROM cases. The x-axis represents the number of days since membrane rupture. (B) Correlation between pGSN levels and WBC counts. Spearman’s rank correlation coefficient was ρ = −0.1874 (95% CI: −0.4689 to 0.2817, p = 0.2289), indicating no significant correlation. pGSN, plasma gelsolin; pPROM, preterm premature rupture of membranes.

**Fig 4:**
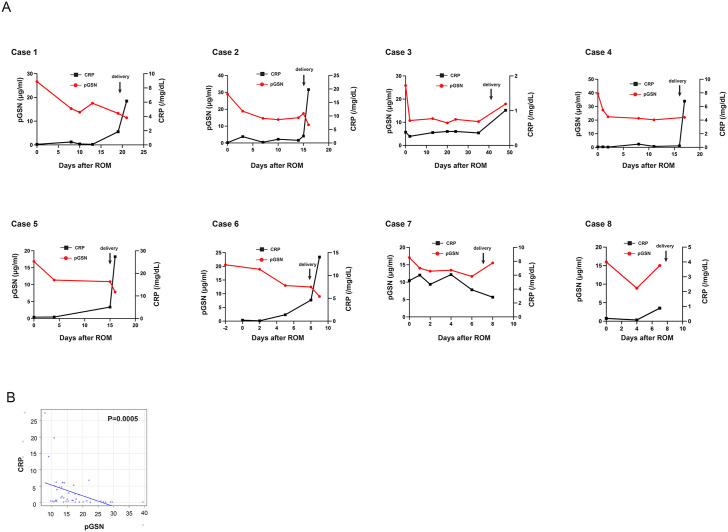
Temporal changes and correlation between pGSN and CRP in pPROM cases. (A) Longitudinal changes in pGSN levels and C-reactive protein (CRP) in individual pPROM cases. The x-axis represents the number of days since membrane rupture. (B) Correlation between pGSN levels and CRP. Spearman’s rank correlation coefficient was ρ = −0.5138 (95% CI: −0.7119 to −0.2400, p = 0.0005), indicating a significant negative correlation. pGSN, plasma gelsolin; pPROM, preterm premature rupture of membranes.

Eight pPROM cases with rupture of membranes between 25 and 32 weeks of gestation were analyzed (Table 1). Among eight gestational age-matched control cases without membrane rupture, six showed histological CAM (stages Ⅱ–Ⅲ), while the remaining two showed no evidence of CAM. As shown in Fig 2A, the mean ± SD pGSN concentrations were 28.09 ± 7.08 μg/mL in the second-trimester group, and 23.53 ± 8.56 μg/mL in women with pPROM. The Mann–Whitney U test revealed no significant difference between the two groups (*p* = 0.16). After the membrane rupture (day 0), pGSN concentrations tended to decrease until delivery (Fig 2B). The rate of pGSN decline positively correlated with the interval from ROM to delivery (Spearman’s ρ = 0.838, *p* = 0.009) (Fig 2C). The correlation between pGSN and WBC was weak and not statistically significant (Figs 3A,3B). In contrast, this decline was observed alongside an increase in CRP levels; a significant negative correlation was identified between pGSN and CRP (Figs 4A and 4B). IL-6 and TNF-α levels were below the detection limit in most cases and were therefore excluded from the analysis (Table 2).

**Table 1:** Clinical characteristics of patients with preterm premature rupture of membranes.

Case	WBC (/μL)	CRP (mg/dL)	IL-6 (pg/mL)	TNF-α (pg/mL)	pGSN (μg/mL) (% reduction from ROM)
1	18200	1.85	undetermined	undetermined	13.2 (50.2)
2	15100	2.57	120.1	undetermined	17.4 (39.6)
3	11700	0.36	undetermined	undetermined	10.2 (60.2)
4	13500	0.14	undetermined	undetermined	20.1 (48.8)
5	14800	5.06	248.4	undetermined	10.8 (35.3)
6	18200	4.61	undetermined	undetermined	12.4 (39.4)
7	12600	3.9	undetermined	undetermined	11.6 (31.6)
8	15000	0.88	undetermined	undetermined	15.0 (5.95)

ANS, antenatal steroid; C/S, cesarean section; CAM, chorioamnionitis; GA, gestational age; GP, gravida para; ROM, rupture of membrane; Umb-A, Umbilical artery

**Table 2:** Peripartum inflammatory markers and pGSN levels.

Case	Age	GP	GA at ROM	GA at delivery	ANS	Days from ROM to delivery	Delivery mode	Birth weight	Apgar score (1’/5’)	Umb-A pH	CAM Stage	Funisitis Stage
1	31	G3P0	25w4d	28w3d	+	20	Em C/S	1258	7/7	7.43	III	III
2	40	G1P0	32w5d	34w6d	–	15	Em C/S	2171	8/8	7.36	III	III
3	35	G3P2	28w1d	34w0d	+	41	Em C/S	1995	8/8	7.33	–	–
4	34	G3P2	26w3d	28w4d	+	14	Em C/S	1121	4/6	7.33	–	–
5	37	G2P1	30w6d	33w0d	+	15	Em C/S	1900	2/6	7.29	III	II
6	31	G2P1	30w6d	32w0d	+	8	Em C/S	1589	8/9	7.32	III	III
7	37	G2P0	24w6d	25w6d	+	7	vaginal	948	5/6	7.55	III	I
8	37	G2P0	33w1d	34w2d	+	8	vaginal	2052	8/9	7.31	II	–

### Histological evaluation of GSN expression in CAM

**Fig 5:**
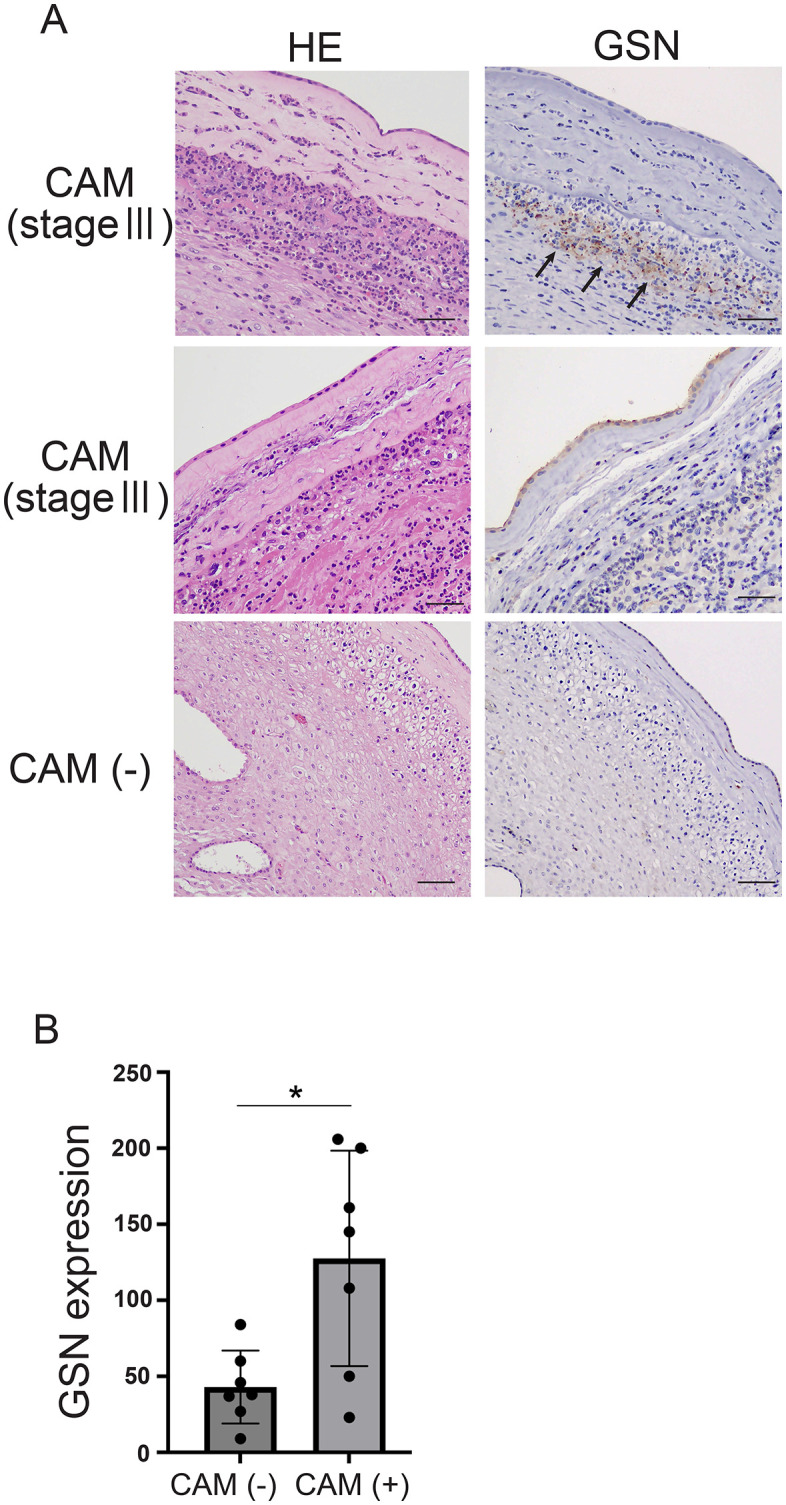
Representative immunohistochemical images of amniotic membranes. (A) In one stage-Ⅲ CAM case, localized expression of GSN was observed in the chorionic membrane layer (arrows). In contrast, no GSN expression was detected in another stage-Ⅲ CAM case or a CAM-negative control. (B) Quantification of GSN-positive cells showed a significantly higher count in the CAM group compared to the non-CAM groups. Scale bar = 50 μm; pGSN, plasma gelsolin; CAM, chorioamnionitis.

Immunohistochemical analysis revealed that GSN-positive cells were sparse in the amnion of non-CAM cases, whereas in CAM stage-III cases, an accumulation of GSN-positive cells was specifically observed in the subchorionic layer of the fetal membranes (Fig 5A). Quantification of GSN-positive cells in 10 randomly selected fields at 200 × magnification showed a significantly higher count in the CAM group (n = 7) compared to the non-CAM group (n = 7; Fig 5B). However, the distribution patterns of GSN-positive cells varied across individual cases, and no consistent correlation with CAM staging was observed (supplementary Table 1).

## Discussion

This study provides a preliminary investigation into the dynamics of pGSN and its association with intrauterine inflammation during pregnancy. pGSN plays a protective role by scavenging toxic filamentous actin released into the bloodstream during cell necrosis or other tissue damage [12], and its levels are known to decrease in acute conditions such as sepsis and trauma [6]. The clinical importance of pGSN has been increasingly recognized in relation to disease severity and outcomes.

We observed a marked decline in pGSN levels during pregnancy, with a gradual decrease toward the third trimester (Fig 1A). However, no clear correlation was observed between pGSN levels in late pregnancy and the number of days to spontaneous vaginal delivery (S1 Fig), suggesting that pGSN may have limited utility as a predictive marker for the timing of labor.

In cases of pPROM, a significant clinical challenge lies in the lack of reliable biomarkers that can accurately predict or assess intrauterine inflammation (e.g., CAM) at an early stage. In current clinical practice, general inflammatory markers such as WBC and CRP are commonly used; however, they are susceptible to physiological changes associated with pregnancy and steroids, limiting early detection of CAM. In the present study, pGSN concentrations at the time of membrane rupture were not significantly different from those in healthy pregnancies, suggesting that a single pGSN measurement may not be sufficient to predict ROM (Fig 2A). However, our longitudinal analysis indicated that pGSN levels declined as delivery approached (Fig 2B), suggesting that dynamic changes rather than absolute values may have greater predictive relevance. Seven out of eight pPROM cases experienced the onset of labor or were diagnosed with clinical CAM and delivered within 21 days after membrane rupture (Table 1,2). In seven of the eight cases of pPROM, pGSN levels decreased by approximately 30% or more between membrane rupture and delivery (Table 2). Although the observed positive correlation (Fig 2C) suggests that a rapid decline in pGSN may indicate imminent delivery following ROM, the analysis was limited by the small sample size (n = 8) and should only be considered hypothesis-generating. These findings suggest that the decrease in pGSN may reflect the progression of intrauterine inflammation; however, further investigation is needed to determine its utility as a predictive marker for the timing of delivery.

The temporal changes in WBC and CRP following membrane rupture in eight pPROM cases did not show a consistent trend. In contrast, pGSN levels exhibited a near-uniform decline from membrane rupture until delivery (Fig 2B), and a significant negative correlation with CRP was observed (Fig 4B). In most cases, plasma IL-6 and TNF-α levels were below the detection limit in this study. Although several studies have demonstrated that elevated levels of IL-6 and TNF-α in the amniotic fluid are associated with clinical or histological chorioamnionitis [13], few reports have described similar relationships in maternal blood. This suggests that these cytokines may not accurately reflect localized intra-amniotic inflammation when measured in the maternal circulation. In contrast, in our study, maternal pGSN levels decreased after membrane rupture, and further declined as delivery approached. These findings imply that pGSN may more sensitively reflect local inflammatory changes associated with pPROM and the progression toward delivery, even when systemic cytokine levels remain low. Taken together, pGSN could serve as a potential early biomarker for detecting subclinical inflammation preceding clinical chorioamnionitis.

Immunohistochemical analysis revealed a trend toward increased cGSN expression in CAM placentas compared to non-CAM placentas. Nevertheless, even among stage-III CAM placentas, GSN expression varied considerably (Fig 5A), and no consistent correlation with histological severity was found. GSN possesses anti-inflammatory and immunomodulatory properties, and its expression is influenced by complex regulatory mechanisms involving tissue injury, actin release, and cytokine signaling. Therefore, GSN expression may not directly correlate with neutrophil infiltration levels used to define the histological stage of CAM. This discrepancy could explain the heterogeneous GSN staining observed even among stage III CAM cases, suggesting that GSN reflects distinct aspects of the inflammatory process beyond neutrophil accumulation. Moreover, pGSN levels decreased after membrane rupture even in cases without histological evidence of CAM (Table 1), suggesting that pGSN could serve as a more consistent and sensitive marker for predicting delivery in pPROM.

A significant strength of this study lies in the sequential monitoring of pGSN levels alongside WBC and CRP in patients with pPROM following membrane rupture. All included cases represent a well-characterized cohort in which pregnancy was terminated either due to the onset of spontaneous labor or due to clinical chorioamnionitis, providing strong support for the relevance of our findings to intrauterine inflammation. Although the relative decrease in pGSN after membrane rupture did not effectively predict the timing of delivery in our cohort (Table 2), the consistent downward trend observed across all cases suggests that pGSN may serve as an auxiliary marker in clinical scenarios where the diagnosis of membrane rupture is uncertain—such as in cases of false ROMs, where the chorion is disrupted but the amnion remains intact [14]. However, to ensure diagnostic specificity, further validation is needed to confirm that pGSN levels remain stable in false ROM cases.

This study has few limitations. First, it was a single-center observational study with a limited number of cases. Second, due to constraints in sample collection, we were unable to analyze longitudinal data from the same patients throughout the course of pregnancy.

Third, many cytokines such as IL-6 and TNF-α were below the detectable limit. Fourth, this study did not include an assessment of the fetal inflammatory response, such as the measurement of inflammatory markers in umbilical cord blood. Evaluating fetal cytokines, particularly IL-6, would provide valuable insight into the relationship between maternal pGSN and the fetal inflammatory response syndrome (FIRS). FIRS is characterized by elevated fetal plasma IL-6 levels and is associated with adverse neonatal outcomes [15]. While fetal samples were not analyzed in this study, future multicenter studies are warranted to determine whether pGSN can be a useful biomarker for predicting FIRS and preterm birth.

In conclusion, pGSN levels gradually decrease from the mid- to late stages of normal pregnancy, but appear to have limited utility as a predictive marker for the timing of spontaneous labor or delivery. In contrast, in cases of pPROM, pGSN levels decreased relatively rapidly after membrane rupture and exhibited a significant inverse correlation with CRP levels. These findings suggest that pGSN may serve as a supplementary biomarker reflecting the progression of intrauterine inflammation following membrane rupture.

## Supporting information

S1 Fig Spearman correlation between pGSN concentration and days to delivery.  The correlation coefficient was ρ = 0.28 (*p* = 0.205), indicating no statistically significant association between pGSN levels and the interval to spontaneous delivery. pGSN, plasma gelsolin.
(PDF)

S2 Table Scoring of immunohistochemical staining for GSN in control and Stage Ⅲ CAM cases. (DOCX)
