# Supplementary figures and images for: Plasma gelsolin as a potential biomarker for intrauterine inflammation in pregnant women with preterm premature rupture of membranes: A pilot study
Source: PLoS One. 2026 Apr 7;21(4):e0346499. doi: 10.1371/journal.pone.0346499 (PMC13056161; doi:10.1371/journal.pone.0346499)

# Supplementary Figure 1

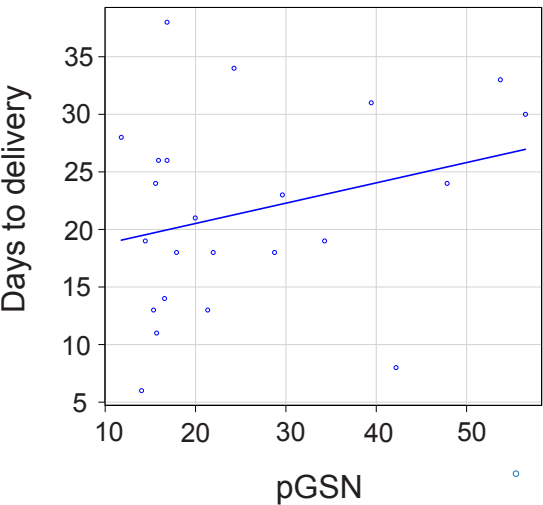

Supplement: S1 Fig — The correlation coefficient was ρ = 0.28 (p = 0.205), indicating no statistically significant association between pGSN levels and the interval to spontaneous delivery. pGSN, plasma gelsolin. (PDF) [file pone.0346499.s001.pdf]
