# Supplementary material for: Plasma gelsolin as a potential biomarker for intrauterine inflammation in pregnant women with preterm premature rupture of membranes: A pilot study
Source: PLoS One. 2026 Apr 7;21(4):e0346499. doi: 10.1371/journal.pone.0346499 (PMC13056161; doi:10.1371/journal.pone.0346499)
Supplement: S2 Table — (DOCX) [file pone.0346499.s002.docx]

**Supplementary Table** Scores of immunohistochemical staining for GSN in control and Stage Ⅲ CAM cases

| Case | Gestational Age  (weeks) | GSN-positive Cells/HPF | CAM stage |
| --- | --- | --- | --- |
| CTR-1 | 35 | 3.8 | None |
| CTR-2 | 27 | 8.4 | None |
| CTR-3 | 28 | 6 | None |
| CTR-4 | 29 | 3.7 | None |
| CTR-5 | 29 | 4.6 | None |
| CTR-6 | 28 | 2.7 | None |
| CTR-7 | 28 | 9 | None |
| CAM-1 | 35 | 2.3 | Ⅲ |
| CAM-2 | 25 | 20 | Ⅲ |
| CAM-3 | 27 | 14.5 | Ⅲ |
| CAM-4 | 26 | 16.1 | Ⅲ |
| CAM-5 | 34 | 10.8 | Ⅲ |
| CAM-6 | 33 | 5 | Ⅲ |
| CAM-7 | 28 | 20.6 | Ⅲ |
